# Comparing 3D ultrastructure of presynaptic and postsynaptic mitochondria

**DOI:** 10.1242/bio.044834

**Published:** 2019-08-15

**Authors:** Thomas Delgado, Ronald S. Petralia, David W. Freeman, Miloslav Sedlacek, Ya-Xian Wang, Stephan D. Brenowitz, Shu-Hsien Sheu, Jeffrey W. Gu, Dimitrios Kapogiannis, Mark P. Mattson, Pamela J. Yao

**Affiliations:** 1 Laboratory of Neurosciences, NIA/NIH, Baltimore, Maryland 21224, USA; 2 Advanced Imaging Core, NIDCD/NIH, Bethesda, Maryland 20892, USA; 3 NIDCD/NIH, Bethesda, Maryland 20892, USA; 4 Janelia Research Campus, Ashburn, Virginia 20147, USA

**Keywords:** Synapse, Dendrite, Axon, FIB-SEM, 3D reconstruction

## Abstract

Serial-section electron microscopy such as FIB-SEM (focused ion beam scanning electron microscopy) has become an important tool for neuroscientists to trace the trajectories and global architecture of neural circuits in the brain, as well as to visualize the 3D ultrastructure of cellular organelles in neurons. In this study, we examined 3D features of mitochondria in electron microscope images generated from serial sections of four regions of mouse brains: nucleus accumbens (NA), hippocampal CA1, somatosensory cortex and dorsal cochlear nucleus (DCN). We compared mitochondria in the presynaptic terminals to those in the postsynaptic/dendritic compartments, and we focused on the shape and size of mitochondria. A common feature of mitochondria among the four brain regions is that presynaptic mitochondria generally are small and short, and most of them do not extend beyond presynaptic terminals. In contrast, the majority of postsynaptic/dendritic mitochondria are large and many of them spread through significant portions of the dendrites. Comparing among the brain areas, the cerebral cortex and DCN have even larger postsynaptic/dendritic mitochondria than the NA and CA1. Our analysis reveals that mitochondria in neurons are differentially sized and arranged according to their subcellular locations, suggesting a spatial organizing principle of mitochondria at the synapse.

## Abstract (precis)

**Summary:** Our 3D ultrastructural analysis of multiple brain regions shows distinctively different sizes and shapes of mitochondria in the presynaptic terminal and postsynaptic dendrite.

## INTRODUCTION

Mitochondria serve many essential functions in the neuron, particularly at the synapse (Harris et al., 2012; Ashrafi and Ryan, 2017). At the presynaptic terminal, mitochondria are central to the operation of neurotransmitter release by supplying highly demanded ATP and by buffering local calcium content (Rangaraju et al., 2014; Ashrafi and Ryan, 2017). Incapacitation of the mitochondria, and the synaptic dysfunction that follows, contribute to or even account for a wide range of neurological defects (Mattson et al., 2008). Postsynaptic mitochondria are also important for synaptic functions, as experimentally disrupting dendritic mitochondria leads dendritic spines and their synapses to fall apart (Li et al., 2004; Schuman and Chan, 2004).

Since the first electron micrograph of mitochondria (Palade, 1952, 1953), electron microscopy (EM) has enlightened us on the anatomy and ultrastructure of mitochondria at a nanoscale resolution. We have learned that the structural appearance of mitochondria is tightly linked to their functional state. Among the many studies that have demonstrated this, Cserép et al. (2018) recently compared the ultrastructure of mitochondria in different types of axons, and found that presynaptic mitochondria in axons of high-activity neurons are larger and contain more densely packed lamellar cristae than those of low-activity neurons.

In a previous EM study, we compared mitochondria between presynaptic and postsynaptic compartments of hippocampal neurons, and noticed that presynaptic mitochondria are smaller and usually darker than postsynaptic mitochondria (Freeman et al., 2017). However, the 2D snapshot views of mitochondria did not allow us to look into their entirety in a spatial context. This is particularly relevant for understanding mitochondria in neurons that have elaborate morphologies including long and convoluted axons and dendrites. Complementing our 2D EM visuals was a 3D EM study, in which the authors revealed that mitochondria in axons of hippocampal neurons are short (less than 3 µm in length), whereas mitochondria in dendrites are long and filamentous (Popov et al., 2005). Here we exploit multiple mouse brain regions that have been serial-sectioned, EM imaged and aligned. We survey mitochondria in or near synapses and focus on analyzing the shape and size of 3D reconstructed mitochondria.

## RESULTS AND DISCUSSION

We examined two populations of mitochondria, presynaptic mitochondria and postsynaptic/dendritic mitochondria. Presynaptic mitochondria are defined by mitochondria located in the presynaptic compartment, which is identified based on the presence of synaptic vesicles, a synaptic cleft and apposing postsynaptic membrane laced with a visible postsynaptic density. Postsynaptic/dendritic mitochondria are defined as those mitochondria in a postsynaptic compartment or dendrite that is identified based on its contact with at least one presynaptic terminal. In this study, postsynaptic/dendritic mitochondria are referred to as postsynaptic mitochondria.

**Fig. 1.**
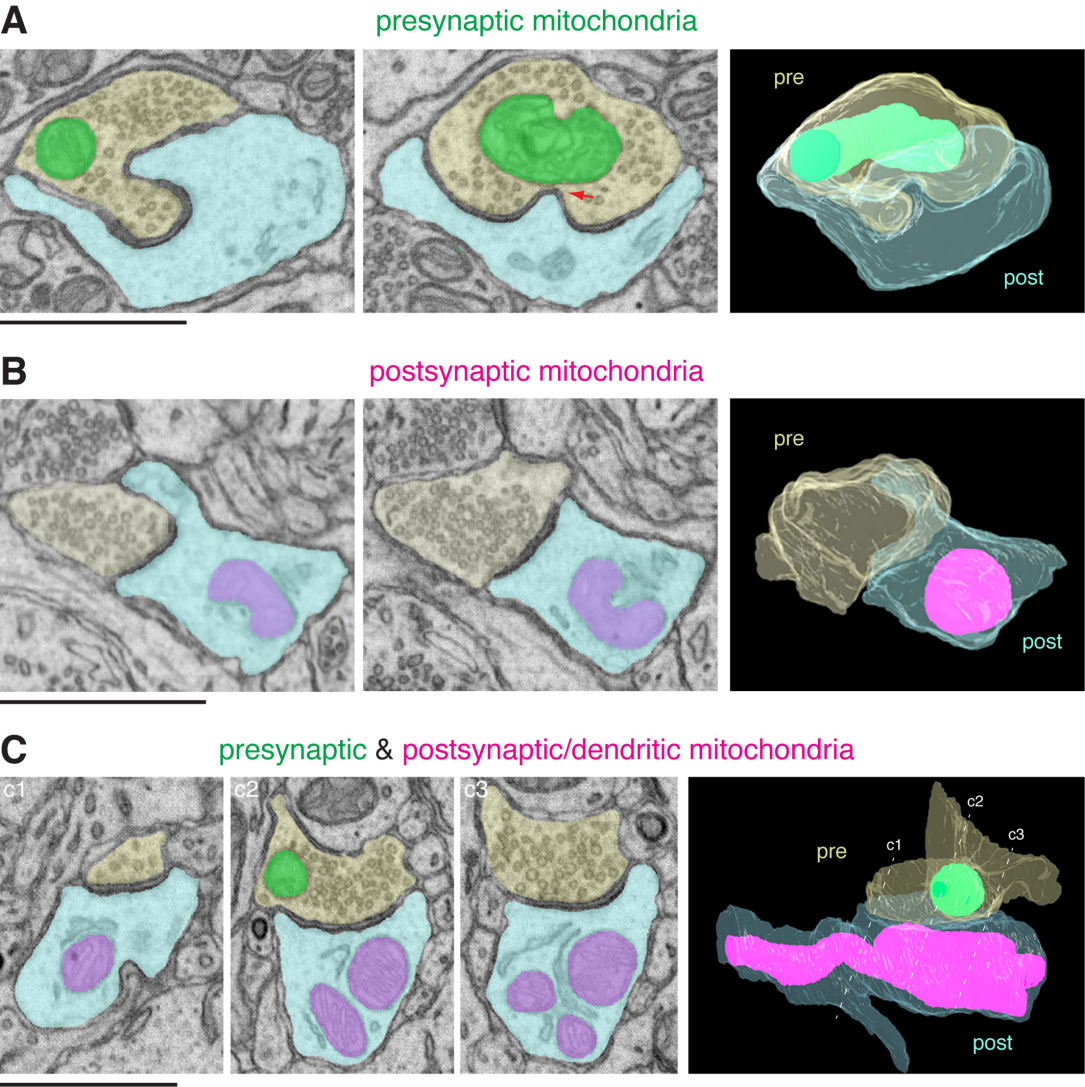
**Synaptic mitochondria (Nucleus accumbens).** (A) Presynaptic mitochondria. Two different 2D EM views of a presynaptic terminal (pale yellow) synapse with a postsynaptic structure (pale blue). A mitochondrion (green) is in the presynaptic terminal only. 3D reconstruction of the mitochondrion and synapse is shown on the right. pre, presynaptic; post, postsynaptic. Red arrow points to a contact between the presynaptic mitochondrion and a postsynaptic membrane invagination. (B) Postsynaptic mitochondria. Two different 2D EM views of a synapse and its 3D reconstruction view. In this synapse, a mitochondrion (magenta/bluish magenta) is in the postsynaptic compartment only. (C) Pre- and postsynaptic mitochondria. Three different 2D EM views of a synapse and its 3D reconstruction view. In this synapse, both pre- and postsynaptic structures contain mitochondria. Dotted lines c1, c2 and c3 in the 3D view approximately correspond to the planes of the three 2D EM images. Note that among the three postsynaptic mitochondrial profiles, two of them are part of a single mitochondrion. In both B and C, note the close contacts between mitochondrion and surrounding endoplasmic reticulum. Scale bars: 1 µm.

We traced all mitochondria in their entirety. Fig. 1A shows an example of a synapse containing a mitochondrion only in the presynaptic compartment, not in the postsynaptic side of the synapse. Fig. 1B shows an example of the opposite: a mitochondrion in the postsynaptic compartment, but not in the opposing presynaptic terminal. Fig. 1C shows mitochondria on both sides of a synapse. Also note in Fig. 1C, the presynaptic mitochondrion is small and stays within the boundary of the presynaptic terminal, whereas the postsynaptic mitochondria are longer and extend beyond the synaptic area.

**Fig. 2.**
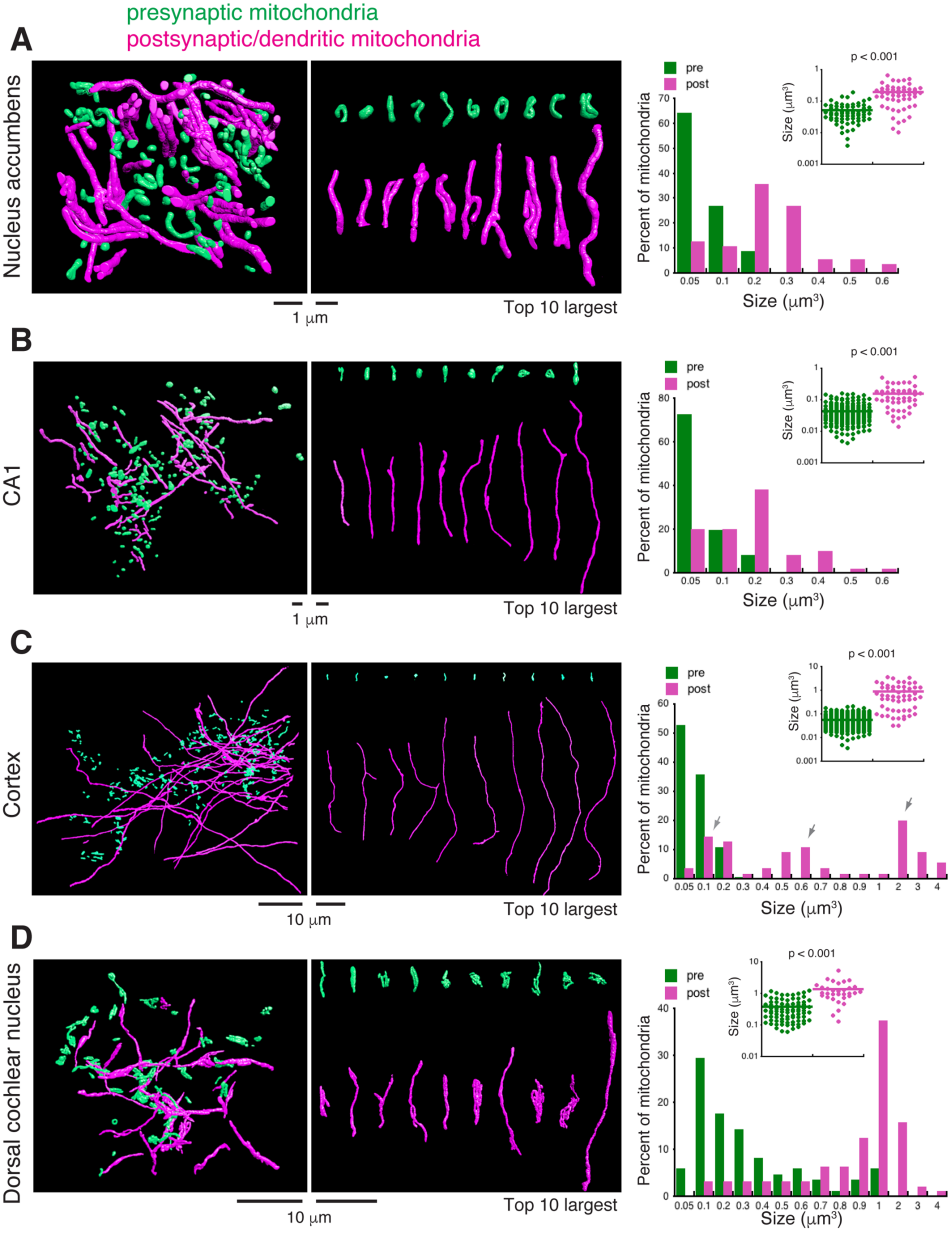
**Comparing presynaptic and postsynaptic mitochondria.** (A) Nucleus accumbens. Left panel shows traced and 3D reconstructed presynaptic (green) and postsynaptic (magenta) mitochondria. Middle panel shows samples of 10 largest presynaptic (green) and postsynaptic (magenta) mitochondria. Right panel shows analyses of size distribution (bar graph) and average size (dot graph) showing that presynaptic mitochondria are significantly smaller than postsynaptic mitochondria (presynaptic mitochondria 0.05 µm^3^±0.004, *n*=67, versus postsynaptic mitochondria 0.195 µm^3^±0.018, *n*=56, *P*<0.001, unpaired *t*-test). (B) CA1. Same as above, left panel shows traced and 3D reconstructed presynaptic and postsynaptic mitochondria; middle panel shows samples of 10 largest presynaptic and postsynaptic mitochondria; right panel shows graphs that show smaller presynaptic and significantly larger postsynaptic mitochondria (presynaptic mitochondria 0.043 µm^3^±0.002, *n*=200, versus postsynaptic mitochondria 0.158 µm^3^±0.017, *n*=50, *P*<0.001, unpaired *t*-test). (C) Somatosensory cortex. Also same as above, left panel shows traced and 3D reconstructed presynaptic and postsynaptic mitochondria; middle panel shows samples of 10 largest presynaptic and postsynaptic mitochondria; right panel shows graphs that show smaller presynaptic and significantly larger postsynaptic mitochondria (presynaptic mitochondria 0.056 µm^3^±0.002, *n*=324, versus postsynaptic mitochondria 0.89 µm^3^±0.124, *n*=55, *P*<0.001, unpaired *t*-test). Notice that postsynaptic mitochondria have three subpopulations (arrows). (D) Dorsal cochlear nucleus. Same as above, left panel shows traced and 3D reconstructed presynaptic and postsynaptic mitochondria; middle panel shows samples of 10 largest presynaptic and postsynaptic mitochondria; right panel shows graphs that show smaller presynaptic and significantly larger postsynaptic mitochondria (presynaptic mitochondria 0.375 µm^3^±0.03, *n*=85, versus postsynaptic mitochondria 1.357 µm^3^±0.182, *n*=32, *P*<0.001, unpaired *t*-test).

We analyzed synapses of four different mouse brain regions: nucleus accumbens (NA), hippocampal CA1, somatosensory cortex and dorsal cochlear nucleus (DCN). The NA dataset consisted of 562 aligned FIB-SEM images with a voxel of 4×4×4 nm (Xu et al., 2017; Wu et al., 2017). We traced all traceable mitochondria on both presynaptic and postsynaptic sides of synapses (67 presynaptic mitochondria, 56 postsynaptic mitochondria). Focusing on comparing their sizes as determined by volume, we found that presynaptic mitochondria are small with a narrow size distribution, whereas postsynaptic mitochondria are significantly larger (Fig. 2A, presynaptic mitochondria 0.05 µm^3^±0.004 versus postsynaptic mitochondria 0.195 µm^3^±0.018, *P*<0.001). We next analyzed hippocampal CA1 neurons. The CA1 dataset consisted of 5080 aligned FIB-SEM images with a voxel of 8×8×8 nm (Gao et al., 2019). We traced 200 presynaptic mitochondria and 50 postsynaptic mitochondria. The size difference between the presynaptic and postsynaptic mitochondria in the CA1 neurons was nearly identical to the difference observed for the NA neurons: significantly smaller presynaptic mitochondria compared to the postsynaptic mitochondria (Fig. 2B, presynaptic mitochondria 0.043 µm^3^±0.002 versus postsynaptic mitochondria 0.158 µm^3^±0.017, *P*<0.001).

**Fig. 3.**
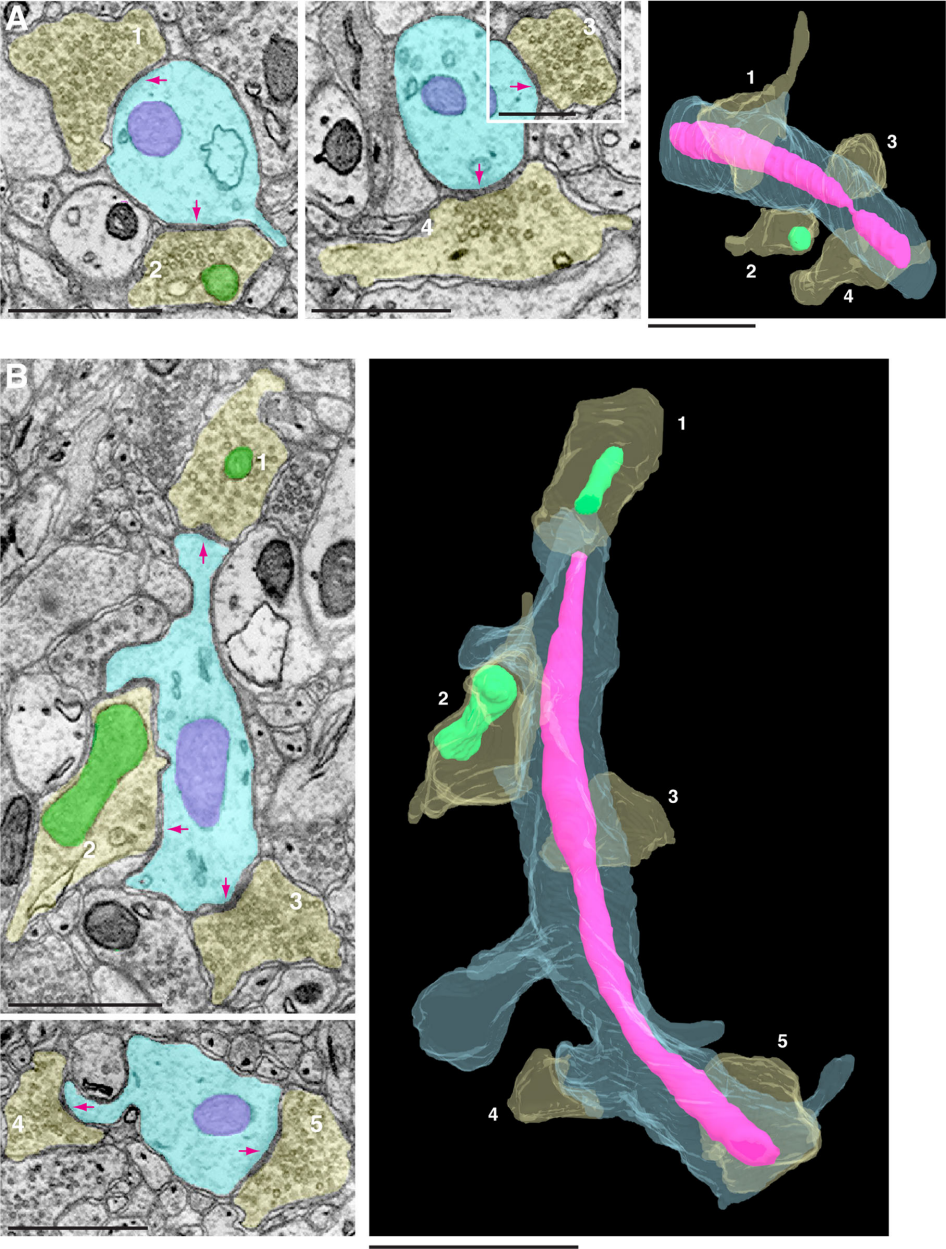
**Dendritic mitochondria span multiple synapses.** (A,B) Examples of two different dendritic mitochondria (magenta), from two separate dendrites (light blue) in the cortex. 3D reconstruction views show that the mitochondrion in example A traverses four presynaptic terminals (yellow), and one of the four has a mitochondrion (green); and the mitochondrion in example B traverses five presynaptic terminals (yellow), two of which have mitochondria (green). Arrows indicate the synaptic contact sites for each presynaptic terminal. Note that for presynaptic terminal 2 in example B, the synaptic contact is mostly evident in an adjacent slice (not shown). Scale bars: 1 µm.

We also analyzed somatosensory cortical neurons. The somatosensory cortex dataset consisted of 1850 serial EM images with a voxel of 6×6×30 nm (Kasthuri et al., 2015). We traced 324 presynaptic mitochondria and 55 postsynaptic mitochondria. Similar to the NA and CA1 neurons, the presynaptic mitochondria of the cortical neurons were significantly smaller than the postsynaptic mitochondria (Fig. 2C, presynaptic mitochondria 0.056 µm^3^±0.002 versus postsynaptic mitochondria 0.89 µm^3^±0.124, *P*<0.001). Among the postsynaptic mitochondria of cortical neurons, closer examination revealed three subpopulations: one group had a size distribution range similar to the size range of postsynaptic mitochondria of the NA or CA1 neurons; the other two groups were 3- to 10-fold larger (arrows in Fig. 2C). Moreover, the larger mitochondria in the dendrites often spanned multiple synapses, as exemplified in Fig. 3.

**Fig. 4.**
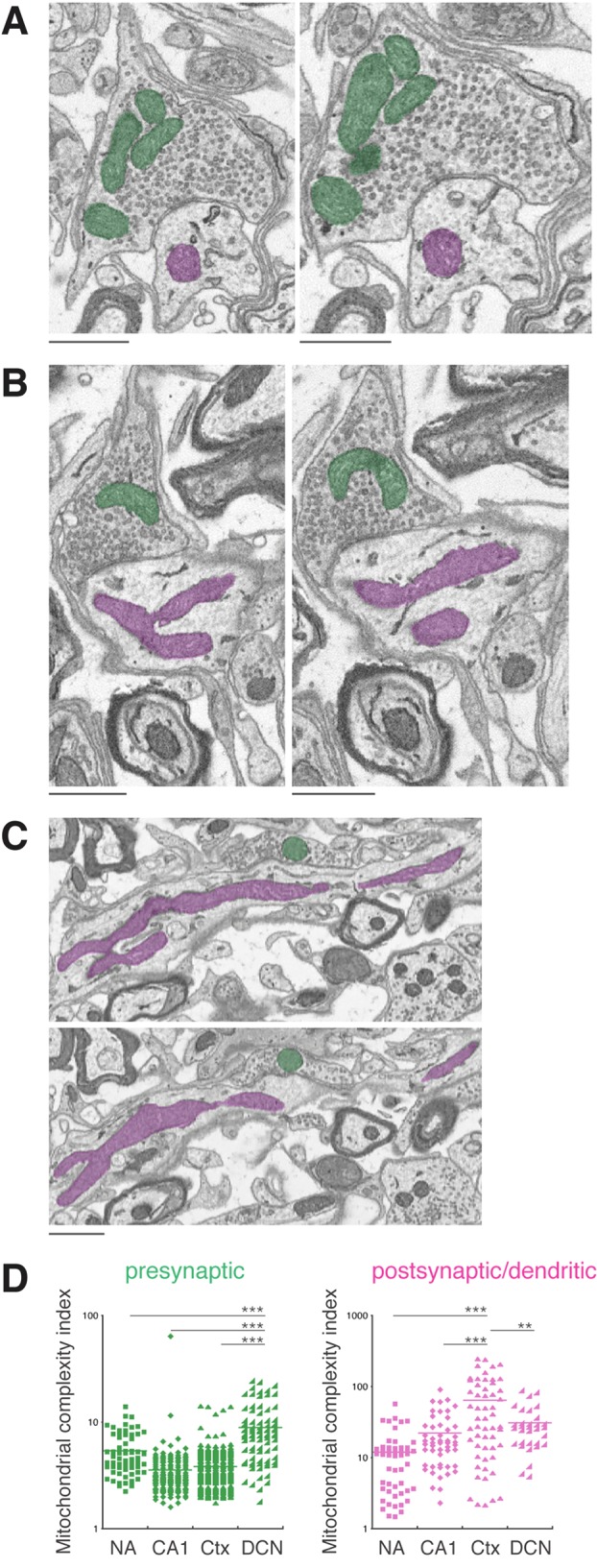
**Greatest complexity is seen in DCN presynaptic mitochondria and cortex postsynaptic mitochondria.** (A–C) EM images from the DCN illustrate the complex arrangements of presynaptic (green) and postsynaptic mitochondria (magenta). Note also several close contacts among presynaptic mitochondria and between postsynaptic endoplasmic reticulum and mitochondria. (D) Mitochondrial complexity indices (MCI) for presynaptic and postsynaptic mitochondria. Most prominent are the high values and wide ranges of the MCIs of the DCN presynaptic mitochondria and cortex (Ctx) postsynaptic mitochondria. ***P<0.001, **P<0.01, unpaired *t*-test. Scale bars: 1 µm.

Finally, we analyzed DCN neurons. Unlike the NA, CA1 and cortex, which were brain tissues from adult mice, the DCN tissue was from a postnatal day 17 young mouse. Also note that DCN is from the hindbrain, while the other three are from forebrain structures. The DCN dataset consisted of 384 serial block-face scanning EM images with a voxel of 8.5×8.5×100 nm. We traced 85 presynaptic mitochondria and 32 postsynaptic mitochondria (Figs 2D and 4A–C). Comparing between the presynaptic and postsynaptic mitochondria, the DCN neurons similarly showed the smaller-presynaptic and larger-postsynaptic mitochondria pattern (Fig. 2D, presynaptic mitochondria 0.375 µm^3^±0.03 versus postsynaptic mitochondria 1.357 µm^3^±0.182, *P*<0.001).

Therefore, one striking similarity among the four brain regions analyzed was that presynaptic mitochondria were always significantly smaller than the postsynaptic mitochondria. However, there were also noticeable differences between the brain regions. Among the presynaptic mitochondria, DCN exhibited a much wider size distribution (histogram in Fig. 2D), and a significantly greater average size compared to all other three brain regions (DCN 0.375 µm^3^±0.031 versus NA 0.05 µm^3^±0.004, *P*<0.001; versus CA1 0.043 µm^3^±0.002, *P*<0.001; versus cortex 0.056 µm^3^±0.002, *P*<0.001). Presynaptic mitochondria of the DCN also showed the greater amount of morphological complexity (Fig. 4D). Among the postsynaptic mitochondria, NA and CA1 had identical sizes (NA 0.195 µm^3^±0.018 versus CA1 0.158 µm^3^±0.017, *P*=0.135), but cortex (0.89 µm^3^±0.124) and DCN (1.357 µm^3^±0.182) were larger and exhibited a greater degree of morphological complexity (Fig. 4D).

**Fig. 5.**
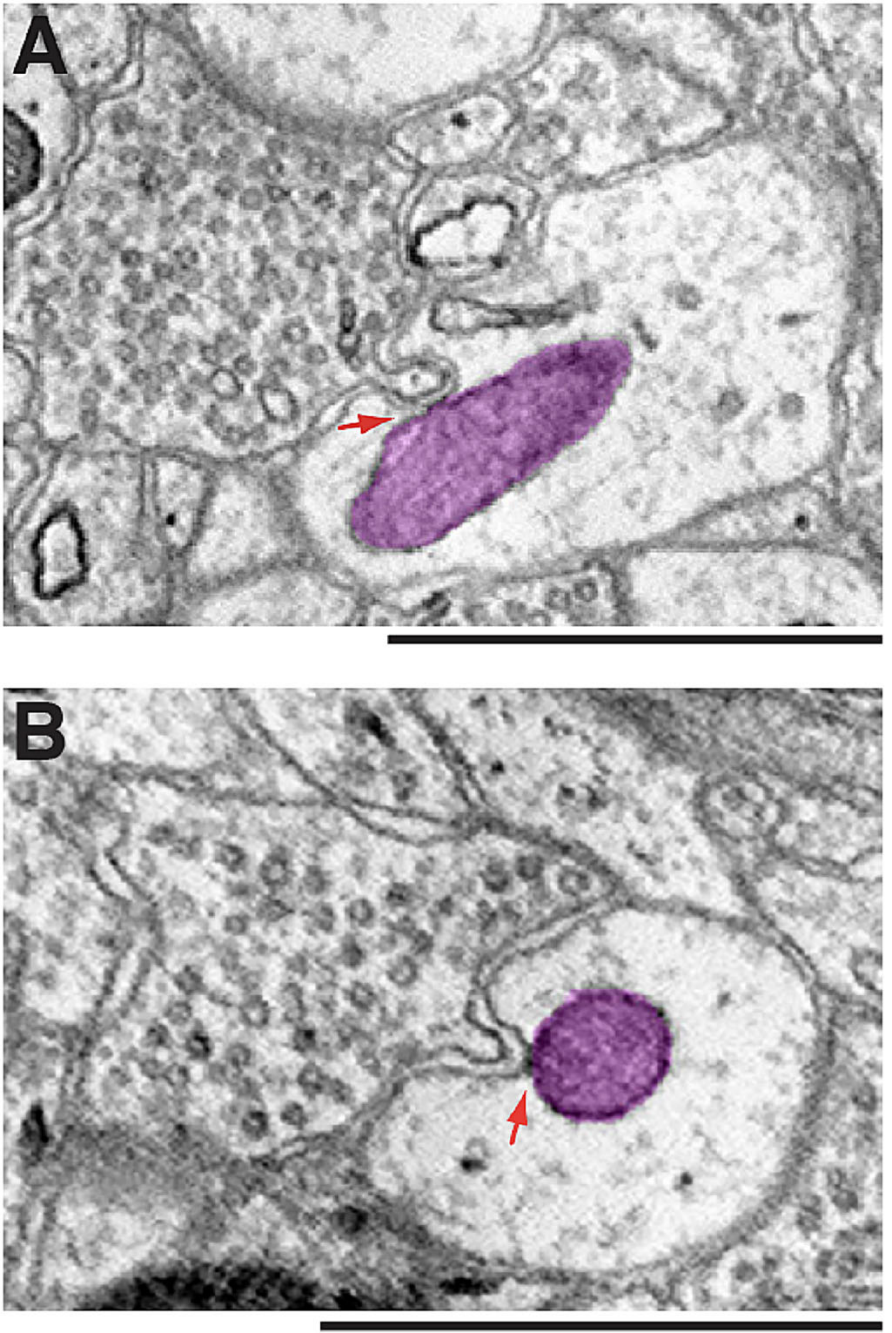
**Mitochondria contacting membrane invaginations.** (A,B) Shown are synapses of somatosensory cortical neurons. In both examples, postsynaptic mitochondria (magenta) make direct contact with presynaptic membrane invaginations (red arrows). Scale bars: 1 µm.

An updated view of cell biology is that cellular organelles do not exist in isolation, but rather they interact with and physically connect to other types of organelles (Valm et al., 2017; Cohen et al., 2018; Guo et al., 2018). For example, mitochondria in neurons have been found to make extensive contacts with the endoplasmic reticulum (Popov et al., 2005; Wu et al., 2017). Consistently, throughout the course of our study, we readily observed frequent contacts between mitochondria and the endoplasmic reticulum (for example, Figs 1B,C and 4A–C) in addition to the contacts between mitochondria themselves (Fig. 4A). We also noticed mitochondria making contacts with synaptic plasma membrane-derived invaginations. Some of these membrane invaginations came from the postsynaptic membrane making direct contact with the mitochondria residing in the presynaptic compartment (Fig. 1A), while other membrane invaginations were in an opposite direction – from the presynaptic membrane protruding into the postsynaptic compartments – and contacting mitochondria there (Fig. 5A,B). A large variety of the membrane invaginations have been seen in neurons of all major groups of animals (Petralia et al., 2015, 2016, 2017, 2018). However, the exact functions of these invaginating membranous structures are not always clear (Petralia et al., 2018). Given that the physical interactions between organelles are believed to serve as portals for exchanging cargo and transmitting molecules such as lipids or calcium (Manford et al., 2012; Valm et al., 2017; Cohen et al., 2018), it seems likely that the contacts between the synaptic membrane invaginations and mitochondria must serve specific functions. Our finding paves a way for future investigations aimed to understand the functions of synaptic membrane invaginations including the mechanisms and functions of their physical interactions with mitochondria.

Overall, our 3D ultrastructural descriptions of the presynaptic and postsynaptic mitochondria are consistent with previous studies of axonal and dendritic mitochondria (Popov et al., 2005; Lewis et al., 2018; Rangaraju et al., 2019) although we concentrated on mitochondria in or near synapses. Additionally, we find that this presynaptic/smaller-mitochondria postsynaptic/larger-mitochondria pattern seems to be a general organizing principle for neuronal mitochondria, regardless of the types of neurons. The report of larger mitochondria in the axons of high-activity neurons than the axons of low-activity neurons (Cserép et al., 2018) implies a positive correlation between the size and activity or function of mitochondria. Nonetheless, it is unclear which specific function of mitochondria is linked to their sizes. A study by Lewis et al. (2018) showed that experimentally enlarging axonal mitochondria did not change mitochondria's ATP production but, instead, enhanced their capacity for Ca^2+^ uptake. Therefore, the consequences of large dendritic mitochondria (Fig. 2) that cover multiple synapses (Fig. 3) may include provision of a large reservoir for Ca^2+^ buffering and integrating inputs from multiple adjacent synapses (Mattson et al., 2008). On the other hand, small mitochondria in presynaptic terminals may enable the large elevation of cytoplasmic Ca^2+^ levels required for release of high amounts of neurotransmitter (Lewis et al., 2018).

In addition to the size difference, our previous 2D ultrastructural analysis also revealed a difference in the intensity: presynaptic mitochondria are darker than postsynaptic mitochondria (Freeman et al., 2017). In the current study, we did not find consistent differences in the intensity between the presynaptic and postsynaptic mitochondria (data not shown). One factor that may contribute to the discrepancy is the type of synapse: mossy terminal synapses of the hippocampal CA3 were analyzed in the previous 2D study (Freeman et al., 2017) whereas synapses of neurons from the NA, CA1, cortex and DCN were analyzed in the current study. For this reason, we surveyed CA3 synapses of a partially constructed dataset (data not shown*)*. While we observed examples of noticeably darker mitochondria in the presynaptic terminals of some of the synapses, the CA3 dataset was not suitable for our intended 3D ultrastructural reconstruction.

## MATERIALS AND METHODS

### EM dataset

The NA FIB-SEM dataset was from previous studies (Xu et al., 2017; Wu et al., 2017). The NA tissue was from the brain of an adult mouse (11-month-old male C57/BL6J). This dataset has 562 slices with a voxel 4×4×4 nm.

Hippocampal CA1 tissue was from a previous study (Gao et al., 2019). The specimen was from the brain of a 2-month-old male mouse (C57/BL6). The FIB-SEM dataset is composed of 5080 serial EM images with 8×8×8 nm voxel resolution.

The somatosensory cortex FIB-SEM dataset was from a previous study (Kasthuri et al., 2015). The somatosensory cortex tissue (layer V or V/VI) was from an adult mouse (BALB/c). The somatosensory cortex dataset consisted of 1850 serial EM images with a voxel of 6×6×30 nm, and the entire dataset is available at https://software.rc.fas.harvard.edu/lichtman/vast/.

DCN tissue was from a postnatal day 17 mouse (CBA/J). Unlike the NA, CA1 and cortex datasets, which were generated by FIB-SEM, the DCN tissue was sectioned using a diamond knife mounted within an SEM. The tissue was prepared using a method modified from Deerinck et al. (2010). Following fixation in 2.5% glutaraldehyde/2% formaldehyde in cacodylate buffer, DCN tissue was washed, incubated in 3% potassium ferrocyanide with 4% osmium tetroxide for 1 h on ice, and then thiocarbohydrazide for 20 min, 2% osmium tetroxide for 30 min and 1% uranyl acetate overnight. The next day, tissue was stained with Walton's lead aspartate for 30 min at 60°C, washed and dehydrated in an ethanol series followed by two changes of propylene oxide and embedded in epon overnight, and finally polymerized in epon for 24 h at 60°C. The embedded tissue was mounted on an aluminum pin using silver-epoxy glue and trimmed, silver paint applied on the sides, and then sputter-coated with gold-palladium. The block was placed in a Zeiss Sigma SEM, and mounted on a Gatan 3View2 ultramicrotome for serial block face imaging. A 70 μm square area, divided into four quadrants, was selected. Sections were cut at 100 nm and the surface was imaged at 2 kV (high current/high vacuum) and 4500×4500×8.5 nm pixels. 955 slices were cut and imaged and the first 501 were selected for more detailed analysis; slices were aligned with Gatan DigitalMicrograph and initial reconstruction studies used IMOD.

### 3D reconstructions

We used VAST (Volume Annotation and Segmentation Tool; Kasthuri et al., 2015; Berger et al., 2018) to label and trace synaptic mitochondria in all datasets. We also traced selected synapses from the NA dataset. The synapses were identified based on the presence of synaptic vesicles, a synaptic cleft, and a clearly visible postsynaptic density. The presynaptic mitochondria were defined as mitochondria in the presynaptic terminal. The postsynaptic mitochondria were defined as mitochondria either in the postsynaptic structure that directly forms a synaptic contact with a presynaptic terminal, or in a dendrite, a part of which can be traced to form a synapse. The traced images and metadata were processed for data analysis in Matlab, followed by 3D reconstructions using 3ds Max (Autodesk).

### Measurements of mitochondria, data analysis and statistics

We used Matlab to analyze the surface area and volume of the traced mitochondria. Mitochondrial complexity index was calculated using a described formula (Vincent et al., 2019). The number of mitochondria measured for each dataset is listed in the figure legend for Fig. 2. Statistical comparisons were performed with KaleidaGraph using unpaired *t*-test. All results are expressed as mean±s.e.m.
